# Expert Consensus Statement: Committee on Implantable Devices and Committee on Social Issues, Japanese Heart Rhythm Society (JHRS) Statement on Remote Monitoring of Cardiac Implantable Electronic Devices

**DOI:** 10.1002/joa3.70437

**Published:** 2026-09-01

**Authors:** Eiichi Watanabe, Nobuhiro Nishii, Akiko Maeda, Yoshiro Sakai, Katsuhito Fujiu, Makoto Suzuki, Ritsushi Kato, Haruhiko Abe, Takashi Noda, Hiroshi Tada, Masahiko Takagi

**Affiliations:** ^1^ Fujita Health University Bantane Hospital Nagoya Japan; ^2^ Okayama University Graduate School of Medicine, Dentistry and Pharmaceutical Sciences Okayama Japan; ^3^ Kyorin University Hospital Mitaka Japan; ^4^ Saiseikai Kumamoto Hospital Kumamoto Japan; ^5^ Graduate School of Medicine, the University of Tokyo Bunkyo City Japan; ^6^ Institute of Science Tokyo Bunkyo City Japan; ^7^ Yokohama Minami Kyosai Hospital Yokohama Japan; ^8^ Saitama Medical University International Medical Center Hidaka City Japan; ^9^ Local Incorporated Administrative Agency Kurate Hospital Kurate Japan; ^10^ Kindai University Hospital Sakai Japan; ^11^ Faculty of Medical Sciences University of Fukui Fukui Japan; ^12^ Kansai Medical University Medical Center Moriguchi Japan

## Abstract

Remote monitoring (RM) has been established as a standard tool for the management of patients with cardiac implantable electronic devices (CIEDs), offering advantages such as early event detection, fewer routine outpatient visits, and reduced mortality. As RM becomes more common, its appropriate implementation and the need for safe, efficient management have become increasingly important. This statement of the Japanese Heart Rhythm Society (JHRS) provides comprehensive guidance for medical professionals and device manufacturers involved in RM. Specifically, it outlines recommendations on personnel placement, workflow optimization, patient and caregiver education, alert notification settings, physiological parameter monitoring, and insurance claims. It also addresses the responsibilities of device manufacturers, the use of third‐party resources, and strategies for reducing workload based on alert notifications. Recommendation classes and evidence levels were determined by the consensus of the writing team, and the proposed recommendations were reviewed and peer‐reviewed by the JHRS' Committee on Implantable Devices.

## Graphical abstract

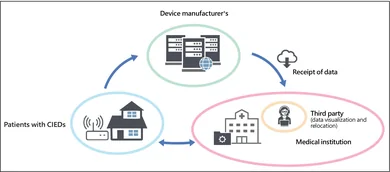

**Abbreviations**
ACC: American College of Cardiology
AHA: American Heart Association
AHRE: atrial high rate episodes
ATP: anti‐tachycardia pacing
CDR: cardiac device representative
CHADS_2_ score: thromboembolic risk prediction score in non‐valvular atrial fibrillation
CIEDs: cardiovascular implantable electronic device
CRT: cardiac resynchronization therapy
CRT‐D: cardiac resynchronization therapy defibrillator
CRT‐P: cardiac resynchronization therapy pacemaker
EOL: end of life
ERI: elective replacement interval
ESC: European Society of Cardiology
HRS: Heart Rhythm Society
ICD: implantable cardioverter defibrillator
ILR: implantable loop recorder
JCS: Japanese Circulation Society
MRI: magnetic resonance imaging
RCT: randomized controlled trial
RI: remote interrogation
RM: remote monitoring
SOP: standard operating procedure

## 1 Introduction

Remote monitoring was approved in the US and EU in 2002 as an effective approach to managing patients with CIEDs. It received regulatory approval in Japan in 2008 and insurance reimbursement in April 2010. Since then, advances have been made in automated data transmission functions, data communications technologies, and security measures. In addition, based on the results of numerous randomized controlled trials (RCTs), RM received a Class I recommendation in a statement issued by the Heart Rhythm Society (HRS) of the US in 2015 and a guideline issued by the Japanese Circulation Society in 2021. Today, RM is regarded as a standard management method for patients with CIEDs that contributes to better quality of care and more timely, appropriate treatment. This statement was developed based on the current situation in Japan, incorporating issues identified over more than a decade of RM management, and also referring to statements issued in the US and Europe. RM includes remote interrogation (RI) and remote monitoring (RM). Remote interrogation is nearly synonymous with manual transmission and refers to systematic device interrogation, while RM involves the automatic transmission of alerts when pre‐specified events occur. At present, RM is available for nearly all types of CIEDs except leadless pacemakers. In this statement, RI and RM, combined, will be referred to as “RM,” but these two terms will be distinguished where appropriate.

## 2 Method of Document Preparation

The writing team, consisting of experts from the JHRS, conducted a comprehensive review of the relevant literature and drafted recommendations based on their findings. The classes of recommendation and levels of evidence were determined in accordance with the latest standards of the American College of Cardiology/American Heart Association (ACC/AHA) (Tables 1 and 2).

**TABLE 1:** Recommendation classes.

Class I
There is evidence and/or general consensus that the evaluation or treatment method is useful and effective.
Class II a
The weight of evidence and/or opinion is in favor of usefulness and effectiveness.
Class II b
Usefulness and effectiveness are less well established.
Class III
There is evidence and/or general consensus that the evaluation or treatment method is not useful and, in some cases, may be harmful.

*Note:* ACC/AHA guideline methodology was used to define recommendation classes. Class I indicates that the benefit clearly outweighs the risk. Class IIa indicates that the weight of evidence favors usefulness. Class IIb indicates that usefulness is less well established. Class III indicates no benefit or potential harm. Colour‐coded in descending order of recommendation strength: green, yellow, pale yellow and orange.

**TABLE 2:** Evidence level.

Level A
High‐quality evidence derived from at least one randomized controlled trial (RCT) A meta‐analysis of data from high‐quality RCTs At least one RCT supported by high‐quality registry‐based studies
Level B‐R (randomized)
Moderate‐quality evidence derived from at least one RCT A meta‐analysis of data from moderate‐quality RCTs
Level B‐NR (non‐randomized)
Moderate‐quality evidence obtained from one or more well‐designed and well‐executed non‐randomized observational studies, observational studies, or registry studies Meta‐analyses of data from these studies
Level C‐LD (limited data)
Evidence from randomized or nonrandomized observational or registry studies with limitations in design or execution Meta‐analyses of data from such studies Physiological or mechanistic studies in humans
Level C‐EO (expert opinion)
Consensus of expert opinion based on clinical experience

*Note:* Evidence levels are defined according to ACC/AHA guideline methodology. Level A indicates evidence derived from multiple randomized controlled trials or meta‐analyses. Level B includes randomized (B‐R) or non‐randomized (B‐NR) studies. Level C includes limited data (C‐LD) or expert opinion (C‐EO). Colour‐coded in descending order of evidence level, with the colours gradually lightening from dark blue to pale blue.

Class I is a strong recommendation, indicating that benefits clearly outweigh risks. Class IIa is a moderate recommendation given when the benefits probably outweigh the risks, while Class IIb indicates that the benefits may be equivalent to or possibly only marginally greater than risks. Class III is a recommendation against a particular action because it provides no benefit and may be harmful. Evidence level A refers to the highest level of evidence, typically derived from multiple randomized controlled trials or a combination of a randomized clinical trial and high‐quality registry data. Evidence level B indicates moderate‐level evidence obtained from a randomized controlled trial (B‐R) or a well‐designed nonrandomized trial (N‐BR). Evidence level C reflects limited evidence and is subdivided into C‐EO (consensus of expert opinion based on clinical experience) and C‐LD (consensus of expert opinion based on limited data). Recommendations with a level of evidence C‐EO reflect pragmatic expert consensus derived from accumulated real‐world experience in remote monitoring practice in Japan, particularly in areas such as workflow organization.

## 3 Evidence That Supports Remote Monitoring

In Japan, the number of patients with heart disease and the number of CIED implantations are increasing with the aging of the population. Cardiac implantable electronic devices are becoming increasingly diverse and complicated due to technological advances in areas such as built‐in sensors, integrated circuits, and communications technologies. Traditionally, follow‐up for patients with CIEDs was completed entirely in the hospital setting. However, with the introduction of RM, the management of these patients has undergone major changes. As a result, the importance of team‐based healthcare in which doctors and other medical professionals collaborate and jointly manage patients is increasing. This statement outlines the clinical evidence supporting RM.

Remote interrogation (RI) refers to scheduled and systematic device interrogation. Most of the data previously obtained through in‐person interrogation at a hospital can now be collected remotely. However, for devices that cannot automatically measure parameters such as pacing threshold, intracardiac signal amplitude, etc., these data cannot be obtained by RM and must be measured in person using a programmer. Remote monitoring refers to the automatic transmission of data based on alerts related to CIED malfunctions or clinical events. Remote monitoring enables early detection of device abnormalities and arrhythmia events, even in asymptomatic patients. Remote interrogation is almost synonymous with manual transmission. Although substantial evidence supports the clinical utility of RM, evidence for RI remains limited. Table 3 shows the RM systems currently available in Japan.

**TABLE 3:** Remote monitoring systems available in Japan.

	Abbott	Biotronik
Remote monitoring system	Merlin.net	Home Monitoring
Name	Merlin@home	CardioMessenger Smart
Appearance	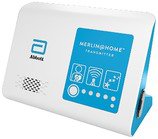	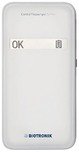
Smartphone app	myMerlinPulse mobile app (ICD and CRT‐D) myMerlin mobile app (ILR)	Incompatible
Compatibility with patient's smartphone	Compatible	Incompatible
Transmitter type	Non‐mobile or smartphone app	Mobile
Communication method	Bluetooth, mobile network, Wi‐Fi, analog phone line, RF	Mobile phone circuit
Transmission frequency	Scheduled transmission, alert event, daily transmission	Daily transmission, alert event, daily communication
Change in transmission frequency program	Possible	Not yet ready
Change in alert and parameter programs	All alert programing possible online (All programmings for alert notification, report setting, data export setting, etc. are possible online. Patients' devices should be set on a one‐by‐one basis.)	Alerts can be set on the website (**)
Transmission by patients	Possible	Not yet possible
Distance to transmitter	Mobile transmitter: < 1.5 m Immobilized transmitter: < 3 m	Within 2 m
Real‐time IEGM at remote follow‐up	30 s	30 s
IEGM of arrhythmic episodes	Save all episodes	Save all episodes
Provider communication method	E‐mail, SMS, phone	E‐mail
FDA and CE Mark approved	Approved	Approved
Additional features	Integrated heart failure website for patients equipped with both CardioMEMS (pulmonary arterial pressure) (*) and Abott CIEDs, compatibility with export to electronic medical charts, patient call‐back function, and CorVue body fluid status alert	Compatibility with export to electronic medical charts, automatic data download function, patient's condition monitoring tool Heart Insight (ICD, CRT‐D)
Remarks	(*): not yet approved in Japan	(**) However, the specification does not allow degrading the importance of some alerts directly related to serious condition or setting them to “off” as a safety function

Boston Scientific	Medtronic	MicroPort
LATITUDE	CareLink	SmartView
LATITUDE NXT Remote Patient Management System	MyCareLink Patient Monitor MyCareLink Relay Home Communicator	SmartView Hotspot SmartView Monitor SmartView Connect (app used to install on communication terminals)
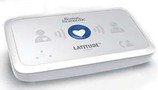	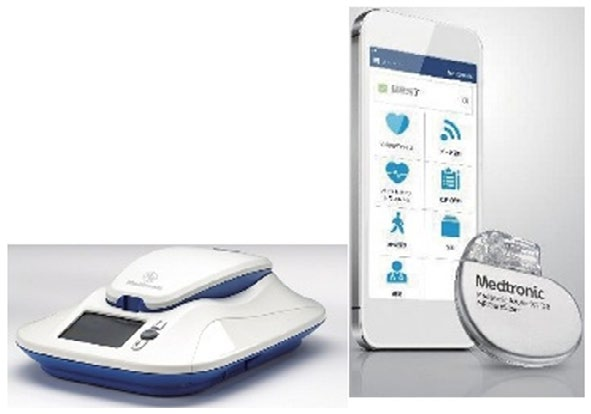	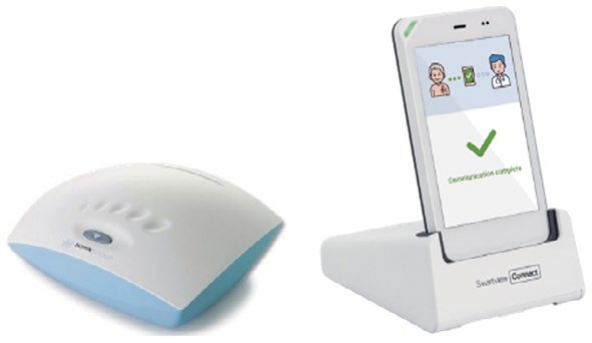
Incompatible	MyCareLink Heart app (ILR, IPG, CRT‐P, ICD and CRT‐D mounted with Bluetooth)	Incompatible
Incompatible	Compatible	Incompatible
Non‐mobile	Non‐mobile or smartphone app	Non‐mobile or mobile
Mobile phone circuit, LAN, Bluetooth (optional)	Bluetooth; mobile phone circuit; Wi‐Fi	Bluetooth; mobile phone circuit
Scheduled transmission, alert event, daily communication	Scheduled transmission, alert event, daily communication	Scheduled transmission, alert event, daily communication
Possible	Possible	Possible
Parameters and alerts can be programed from LATITUDE website	Face‐to‐face programming of parameters and alerts Remote programming possible for parameters, alerts and notifications only in the case of LINQ II.	Face‐to‐face programming of parameters and alerts. Allert notification can be set online.
Possible	Possible	Possible
Within 3 m; handle within the reach of patients in the case of S‐ICD.	Within 3 m	Within 2 m
30 s	10 s	7 s
Save all episodes	Save all episodes	Save all episodes
E‐mail, fax	E‐mail, SMS, website	E‐mail, SMS, fax
Approved	Approved	Approved
Compatibility with export to electronic medical charts, HeartLogistic monitoring, and possibility to set data transmission to healthcare professionals.	Compatibility with export to electronic medical charts: OptiVol Thorax impedance alert: Cardiac Compass HF report	Compatibility with export to electronic medical chart

*Note:* Information reflects device specifications available in Japan as of August 2024. RM indicates automatic transmission of device data and alerts. Compatibility and programming capabilities may vary according to device model and software version.

### 3.1 Benefits of Remote Interrogation

The initial purpose of RI was to manage patients with implantable cardioverter‐defibrillators (ICDs) and reduce the frequency of outpatient visits. In two prospective studies [1, 2], RI was evaluated from the perspectives of both patients and physicians. These studies demonstrated that patients reported high satisfaction with RI, while physicians confirmed that the RI data were reliable, allowing them to fully evaluate the functions of CIEDs and detect arrhythmias while reducing the frequency of outpatient visits [1, 2]. The PREFER study demonstrated that frequent, scheduled RI of pacemakers was superior to scheduled outpatient visits in detecting clinically important findings at an early stage such as ventricular arrhythmia, atrial fibrillation (AF), device abnormalities, lead abnormalities, and battery depletion [3]. Table 4 lists current devices that require RI.

**TABLE 4:** List of devices that require manual transmission by patients themselves (remote interrogation).

Company	
Abbott	None
Biotronik	None
Boston Scientific	PM…… INGENIO ICD……TELIGEN, INCEPTA, EMBLEM CRT‐P……INVIVE CRT‐D……COGNIS, INCEPTA
Medtronic	PM……Advisa, Adapta, Attesta, Micra (both VR + AV), Versa, Sensia, Sphera CRT‐P……Viva, Consulta
MicroPort	PM……KORA250, ENO

*Note:* As of August 2024, leadless pacemakers come only with remote interrogation functions, and not with remote monitoring functions such as automatic alerts.

Abbreviations: CRT‐D, cardiac resynchronization therapy defibrillator; CRT‐P, cardiac resynchronization therapy pacemaker; ICD, implantable cardioverter defibrillator; PM, pacemaker.

### 3.2 Usefulness of Combining Remote Interrogation and Remote Monitoring

The combined use of RI and RM enables continuous, comprehensive RM. This approach allows for daily self‐diagnosis and automatically transmits alerts when abnormalities are detected according to preprogrammed notification criteria.

### 3.3 Patient Follow‐Up Optimization and Patient Safety

**FIGURE 1:**
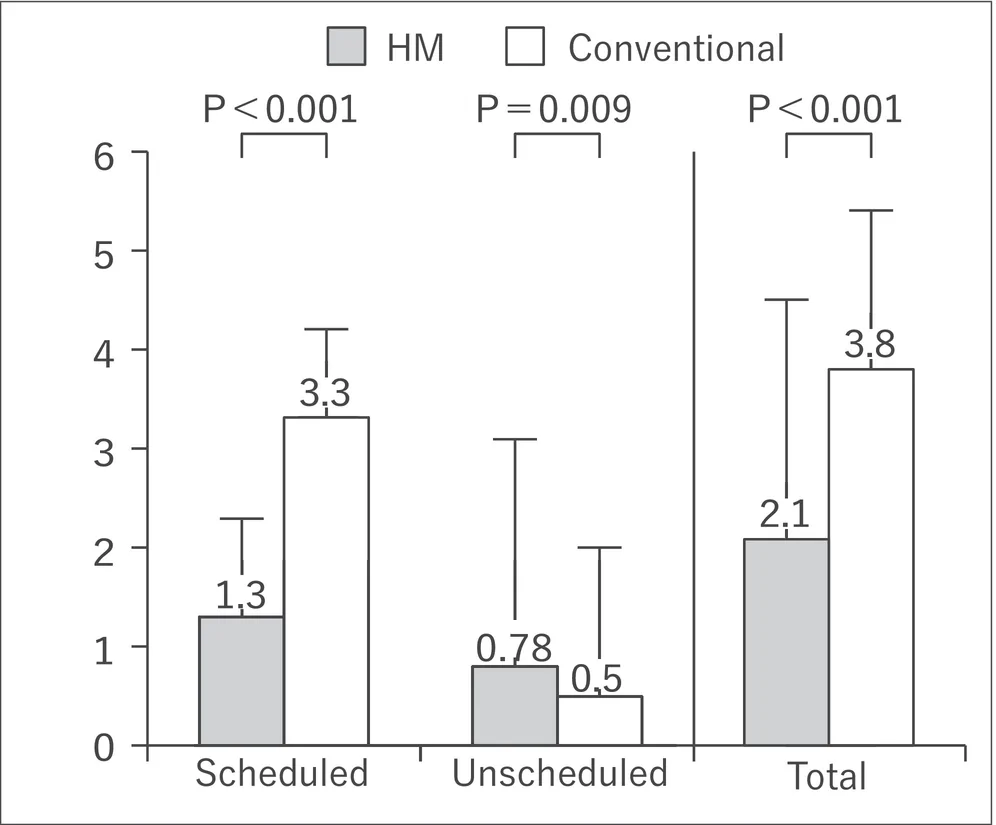
TRUST Study (number of office visits) Patients with ICDs were randomized to the ambulatory treatment group (conventional) and RM group at the ratio of 1:2. Device data were automatically transmitted every day in the RM group, while patients in the ambulatory treatment group were examined in person 4 times a year. Both the number of scheduled visits and the number of unscheduled visits decreased by around 50% in the RM group compared to the ambulatory treatment group. HM: Home monitoring [NB: HM (Remote Home Monitoring) is a trademark of Biotronik Company. It is referred to as HM in figures in Literature 4. Functionally, it is synonymous with RM.] [Extracted from Literature 4].

**FIGURE 2:**
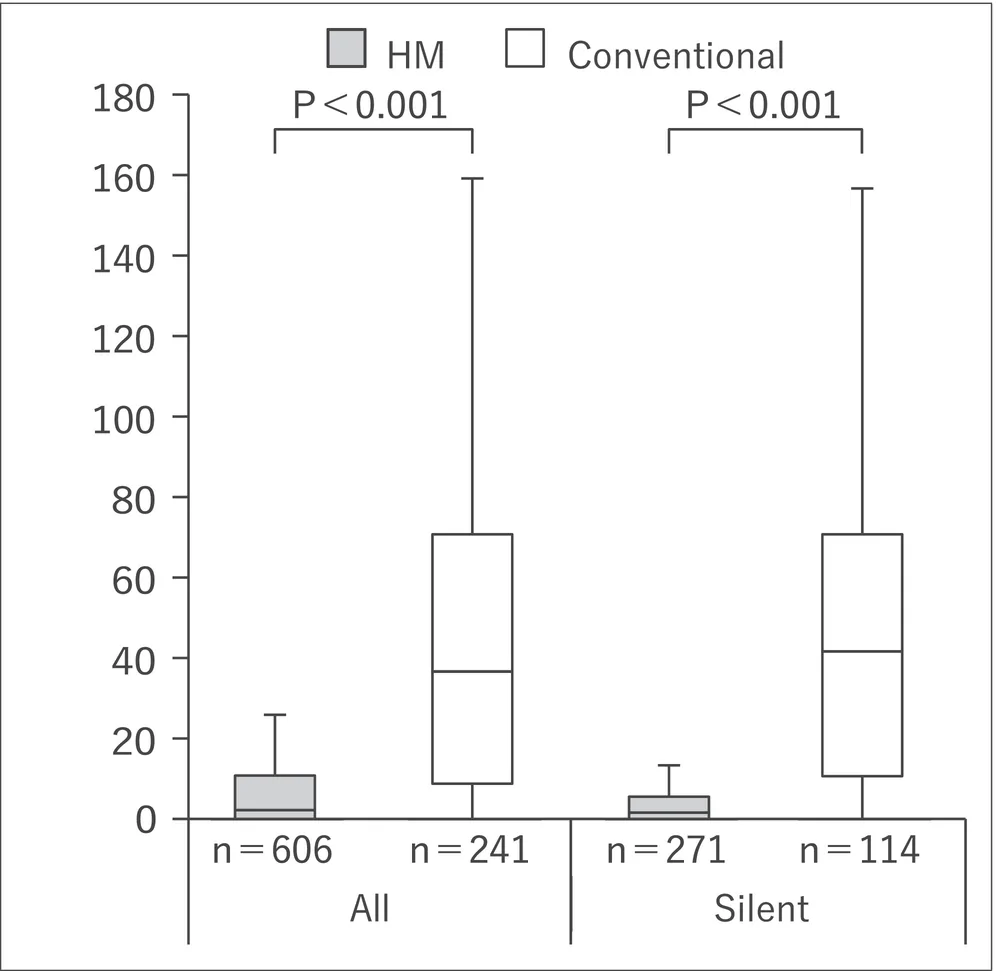
TRUST Study (Number of days from detection of arrhythmia events to intervention) The number of days from the detection of initial atrial fibrillation, ventricular tachycardia, and ventricular fibrillation to medical intervention (All in the figure) was significantly reduced in the RM group compared to the ambulatory treatment group (median: 1 day vs. 35.5 days, *p* < 0.001). In addition, asymptomatic arrhythmia events (initial atrial fibrillation, ventricular tachycardia, ventricular fibrillation, and supraventricular tachycardia) (Silent in the figure) were significantly shortened in the RM group compared to the ambulatory treatment group (median: 1 day vs. 41.5 days, *p* < 0.001). Refer to Figure 1 for abbreviations. [Extracted from Literature 4].

In the TRUST study (2010) [4], patients with ICDs were randomly divided into an outpatient follow‐up group or an RM group in a 1:2 ratio. The RM group did not experience an increased incidence of adverse events, including death, stroke, or surgical procedures, compared to the outpatient group, while the frequency of scheduled and unscheduled hospital visits was reduced by approximately 50% (Figure 1). In addition, median time to the detection of arrhythmia events was shortened to 1 day in the RM group, compared to more than 30 days in patients who underwent in‐person consultations four times per year (Figure 2).

In the CONNECT study [5], the median time from the onset of a clinical event to a clinical decision was reduced from 22 days in the outpatient group to 4.6 days in the RM group. The ECOST study [6] confirmed the long‐term safety of RM over a 24‐month follow‐up period. In the EVOLVO study [7], the RM group showed a 35% lower rate of emergency visits than the standard follow‐up group, and the combined rate of visits for heart failure, arrhythmia, and ICD‐related events was reduced by 21%. In patients with pacemakers, the PREFER [3] and COMPAS studies [8] showed favorable results in the RM group in terms of early event detection and reduced outpatient burden. In Japan, At‐Home study [9] reported that patients in the RM group required outpatient visits only once every 2 years, safely reducing visit frequency to one‐quarter that of conventional follow‐up. Collectively, these large‐scale, multi‐regional prospective studies have consistently shown that RI and RM can replace most routine outpatient visits, decrease the overall number of medical visits, and facilitate early detection of clinically actionable events without compromising patient safety. The principal results of RCTs reported to date are shown in Table 5.

**TABLE 5:** Main findings obtained regarding remote monitoring in RCTs.

Title	Yr	Study type	*N*	Device	Conclusion
PREFER [3]	2009	Multicenter RCT	897	Pacemaker Medtronic CareLink RM	Time to the event detection was shorter in the RM group.
COMPAS [8]	2011	Multicenter RCT	538	Pacemaker Biotronik HM	RM safely reduces the number of device visits. Time to event detection was shorter in the RM group.
At‐Home [9]	2020	Multicenter RCT	1274	Pacemaker Biotronik HM	RM could safely increase the ambulatory visit interval to 2 years. Annual cost was lower in the RM group.
TRUST [4]	2010	Multicenter RCT	1339	ICD Biotronik HM	RM could safely reduce the number of device visits. Time to event detection was shorter in the RM group.
CONNECT [5]	2011	Multicenter RCT	1997	ICD or CRT‐D Medtronic CareLink RM	Time to event detection was shorter in the RM group. The admission period was shorter in the RM group.
ECOST [6]	2012	Multicenter RCT	433	ICD Biotronik HM	RM could safely reduce the number of device visits. RM could reduce the number of appropriate or inappropriate shocks.
EVOLVO [7]	2012	Multicenter RCT	200	ICD or CRT‐D Medtronic CareLink RM	The number of emergent visits was smaller in the RM group. Annual cost was lower in the RM group.
IN‐TIME [10]	2014	Multicenter RCT	716	ICD or CRT‐D Biotronik HM	The number of total deaths and the number of cardiac deaths were smaller in the RM group. RM did not reduce the number of visits for heart failure.

*Note:* Conclusions summarize the primary findings reported in each trial.

Abbreviations: HM, home monitoring; RCT, randomized controlled trial; RM, remote monitoring.

### 3.4 Patient Satisfaction and Quality of Life

Patient acceptance is essential for the successful implementation of RM. Studies evaluating patient satisfaction have reported no significant difference between the RM and the conventional in‐person follow‐up groups in terms of quality of life or satisfaction of care [6, 11, 12]. In the TRUST study, no patients in the RM group crossed over to conventional follow‐up, and 98% chose to continue RM at the end of the study, indicating a high level of patient acceptance and trust in this technology [4].

### 3.5 Device Monitoring

Abnormalities related to CIEDs and leads are rare; however, lead damage and premature battery depletion can lead to life‐threatening complications and require prompt intervention. Remote monitoring alerts medical professionals to lead or device malfunction, but if RM is not in use, these alerts may remain unnoticed until the next scheduled outpatient visit or RI for several months after onset [4]. Lead impedance trends, the number of mode switch actuation events, ventricular arrhythmias, and changes in R‐wave and P‐wave amplitude may be precursors to adverse events, so early detection of these is desirable, and RM makes this possible. Many noise events observed in early stages of lead damage may be mistakenly diagnosed as arrhythmia events, so detailed intracardiac waveform analysis is important for early detection [13]. Remote monitoring is the recommended follow‐up method for patients with CIEDs under safety advisory status. Although increasing the frequency of in‐person visits can aid in early detection, it is cumbersome and inefficient. In contrast, RM enables accurate, efficient, and timely identification of abnormal parameters while optimizing the use of patient, device, and clinical resources.

### 3.6 Shock Reduction

The ability of RM to detect clinical events such as tachycardiac AF, T‐wave oversensing, electromagnetic interference, and device malfunctions allows earlier intervention and reduces the inappropriate ICD shock risk [14, 15, 16]. Even in cases of appropriate ICD shock therapy, RM may result in earlier intervention and a reduction in the total number of treatments. The ECOST study [6, 17], which evaluated ICD shock therapy events as a pre‐specified secondary endpoint, reported a significant reduction in inappropriate therapy in patients assigned to the RM group. The 27‐month rate of inappropriate shock was 10.4% in the conventional follow‐up group versus 5.0% in the RM group. Furthermore, the RM group experienced a lower incidence of all causes of inappropriate shock therapy, including supraventricular tachycardia, noise oversensing, lead abnormalities, and T‐wave oversensing.

### 3.7 Optimizing Device Life

Remote monitoring can help prevent premature CIED battery depletion by enabling early detection of events such as frequent capacitor charging [6, 15, 18]. An increase in the frequency of anti‐tachycardia pacing or premature ventricular contractions may trigger unnecessary shock, which could lead to early intervention to avoid inappropriate shock therapy and preserve battery life. Early detection of atrial tachycardia (AT) or AF also facilitates device reprogramming to adjust settings and avoid inappropriate shock therapy. Because frequent shock delivery significantly shortens battery life, RM contributes to prolonging device longevity by providing timely notifications and preventing therapy interruptions caused by unrecognized events or inappropriate program resets.

### 3.8 Atrial Fibrillation

Remote monitoring has been shown to enable early detection and quantification of AF episodes and overall arrhythmia burden [5, 19, 20]. An analysis of the global Home Monitoring database [21], which included 11 624 patients with pacemakers, ICDs, or cardiac resynchronization therapy defibrillators (CRT‐D), reported a total of 3 004 763 alerts. More than 60% of alerts from pacemakers and CRT‐Ds and approximately 10% of alerts from dual‐chamber ICDs were due to AF. The sensitivity of RM for detecting true AF is approximately 95% [16], and about 90% of AF episodes detected through RM were asymptomatic [20]. Moreover, AF was detected 1 to 5 months earlier in patients with pacemakers in the remote follow‐up group, even when non‐wireless RM systems were used [3]. Early detection of AF may allow early anticoagulation therapy in appropriate patients, potentially preventing cerebral infarction. In addition, early recognition of AF can help avoid inappropriate ICD therapy, predict reductions in CRT pacing percentage, and allow intervention before worsening heart failure occurs.

### 3.9 Stroke Risk

Multiple large‐scale clinical trials have consistently demonstrated an association between AF detected by CIEDs and thromboembolic events [22, 23, 24, 25]. Even short AF episodes lasting as little as 5 min increase the risk of thromboembolic events, and the risk rises with longer episode duration [26, 27]. However, no clear temporal relationship between AF and thromboembolic events has been reported, and no AF episodes were recorded during the 30 days preceding a thromboembolic event [24, 28]. Although risk stratification may be possible by combining AF burden and CHADS_2_ score [26, 27], no specific cutoff line for initiating anticoagulant therapy for AF detected by RM has yet been established. In an intervention study where anticoagulant therapy was initiated or discontinued based on AF burden detected by RM [29], no differences were observed in either stroke incidence or all‐cause mortality. Recently, two randomized controlled studies examined the management of subclinical AF detected episodes detected by CIEDs [30, 31]. The ARTESIA study included patients with asymptomatic AF lasting from 6 min to 24 h and at risk of stroke [30]. The study showed that apixaban significantly reduced the risk of cerebral infarction and systemic embolism compared to aspirin (hazard ratio: 0.63; 95% confidence interval: 0.45–0.88). However, the incidence of major bleeding was higher than with aspirin. The NOAH‐AFNET6 study compared edoxaban with placebo for a composite of endpoints (stroke, systemic embolism, and cardiovascular death) and major bleeding in patients with atrial high rate episodes (AHRE) and multiple stroke risk factors. Edoxaban did not significantly reduce the composite endpoints (hazard ratio: 0.81; 95% confidence interval: 0.60–1.08) and was associated with more frequent major bleeding events [31].

### 3.10 Prediction of Heart Failure Worsening

**FIGURE 3:**
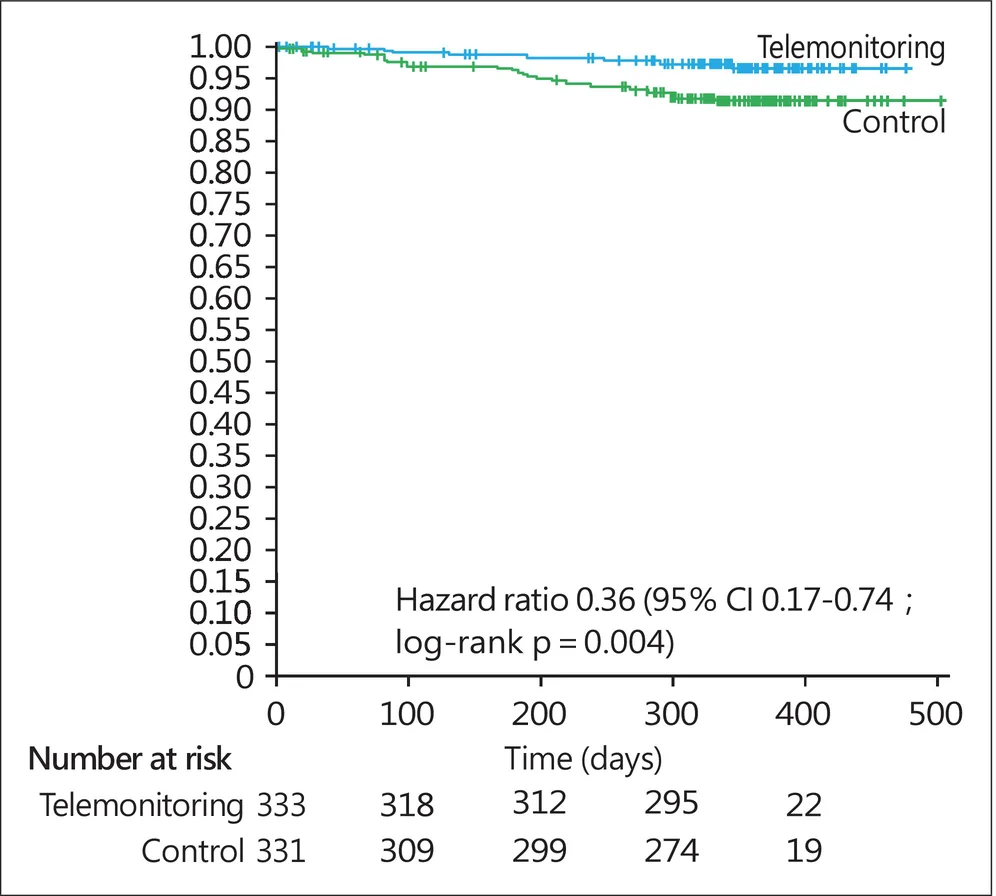
IN‐TIME study The IN‐TIME study enrolled chronic heart failure patients of NYHA class II to III with an ejection fraction of ≤ 35% who recently had a dual chamber ICD or CRT‐D implanted. Patients were randomized into a control group given the standard treatment and an intervention group given the standard treatment plus RM at a ratio of 1:1 and followed up to 12 months. The primary endpoint was a composite clinical score that combined all‐cause deaths, admission of 1 day or longer due to heart failure, changes in NYHA class, and overall assessment by patients themselves. Data were submitted to intention‐to‐treat analysis. Daily RM showed significant improvement of clinical outcome in heart failure patients. [Extracted from Literature 10].

There has been considerable interest in research on predicting the worsening of heart failure using data obtained from CIEDs. Intrathoracic impedance, calculated between the atrial or ventricular lead and the device, has been reported to correlate with intracardiac pressure and body fluid accumulation [32, 33]. Although several non‐randomized/case–control studies have suggested that intrathoracic impedance monitoring may aid in the management of heart failure, randomized controlled studies have reported that such monitoring did not reduce heart failure–related hospitalizations [34, 35]. Other physiological parameters that can be derived from CIEDs include arrhythmia events, heart rate variability, activity level, heart sounds, respiratory rate, and CRT pacing percentage. Comprehensive assessment of these parameters has been reported to enable early prediction of patients at high risk for heart‐failure hospitalization [36, 37, 38]. In the IN‐TIME study [10], daily automatic RM enabled early clinical response to alerts related to worsening heart failure, which resulted in reduced rates of all‐cause and cardiovascular mortality (Figure 3). In addition, the MultiSENSE study [38] evaluated the usefulness of the HeartLogic Index, which integrates five physiological indicators (heart sounds, intrathoracic impedance, pulse rate, respiratory rate, and activity level) for predicting the worsening of heart failure. A HeartLogic Index score of 16 was found to have a sensitivity of 70% and a false‐positive rate of 1.47 people/year, suggesting its utility in identifying patients at risk for worsening of heart failure. Similar findings were reported in Japan in a subanalysis of the HINODE study [39].

### 3.11 Implantable Loop Recorder

Implantable loop recorders (ILRs) play an important role in detecting infrequent arrhythmias and diagnosing the causes of syncope. Important diagnostic information can be obtained within the first few days after ILR implantation [40]. To avoid missing this information, it is important to initiate RM as early as possible after implantation. In patients with stroke of undetermined cause, studies have shown that the combination of ILR and RI detects AF more frequently than conventional long‐term electrocardiographic monitoring [41]. However, because ILRs have limited recording capacity, important diagnostic data can be lost if data are overwritten. ILRs equipped with RM capability can overcome this limitation by automatically transmitting stored data and thereby facilitating earlier diagnosis. For patients using RI, education before implantation is essential so that they can transmit data manually or activate the event button immediately after an episode of syncope, ensuring that critical data are captured and preserved.

### 3.12 Synopsis

Numerous clinical studies have demonstrated the effectiveness of RM and RI in patients with CIEDs. Remote monitoring refers to the automatic transmission of device data, while RI refers to the periodic transmission of device data initiated by patients. In this Statement, these two are collectively defined as Remote monitoring. Through early detection of asymptomatic device abnormalities and arrythmia events in particular, RM can reduce the number of outpatient visits while ensuring patient safety. Combined use of RM and RI allows for continuous monitoring, which can reduce inappropriate shocks, assist in predicting worsening of heart failure, and extend device longevity. Studies have also shown that patient satisfaction with RM is high, and that RM does not adversely affect quality of life. Based on this accumulated evidence, RM is recommended as an effective strategy for managing patients with CIEDs. The corresponding level of evidence for RM is summarized in Table 6 [42].

**TABLE 6:** Level of evidence for conducting remote monitoring [42].

Recommendation class	Evidence level	Recommendation
I	C‐EO	Initiate RM promptly after implantation in ILR‐implanted patients [40, 43].
IIa	B‐NR	Consider initiating RM within 2 weeks before discharging or implantation in CIED‐implanted patients [44, 45].
IIa	A	Consider implementing RM for all CIED‐implanted patients [43].
IIa	A	Consider initiating monitoring of physiological parameters to predict worsening of heart failure in CIED‐implanted patients for heart failure management [38].

*Note:* Recommendations are based on consensus review of available clinical evidence and prior international guidelines including HRS/EHRA/APHRS/LAHRS expert consensus statements. Colour‐coded in descending order of recommendation strength (green, yellow, pale yellow, orange) and evidence level (dark blue gradually lightening to pale blue).

## 4 Patient Education for the Introduction and Management of Remote Monitoring

The introduction of an RM system is recommended as a standard management method after CIED implantation. When RM is initiated, it is important to explain the following points to patients, their families, and caregivers [42, 43].

### 4.1 In‐Person Consultation and Remote Monitoring Systems

Patients with CIEDs should be informed that RM is highly useful and are recommended as a standard management tool [42, 43]. The timing of introducing RM should be considered based on the patient's understanding and acceptance, although earlier initiation (within 2 weeks after implantation for instance) is preferable [44]. Other appropriate opportunities to introduce RM include device replacement or product recall. Even when RM is implemented, at least one in‐person consultation a year remains desirable [42].

### 4.2 Clinical Usefulness of Remote Monitoring and Their Recommendation

**FIGURE 4:**
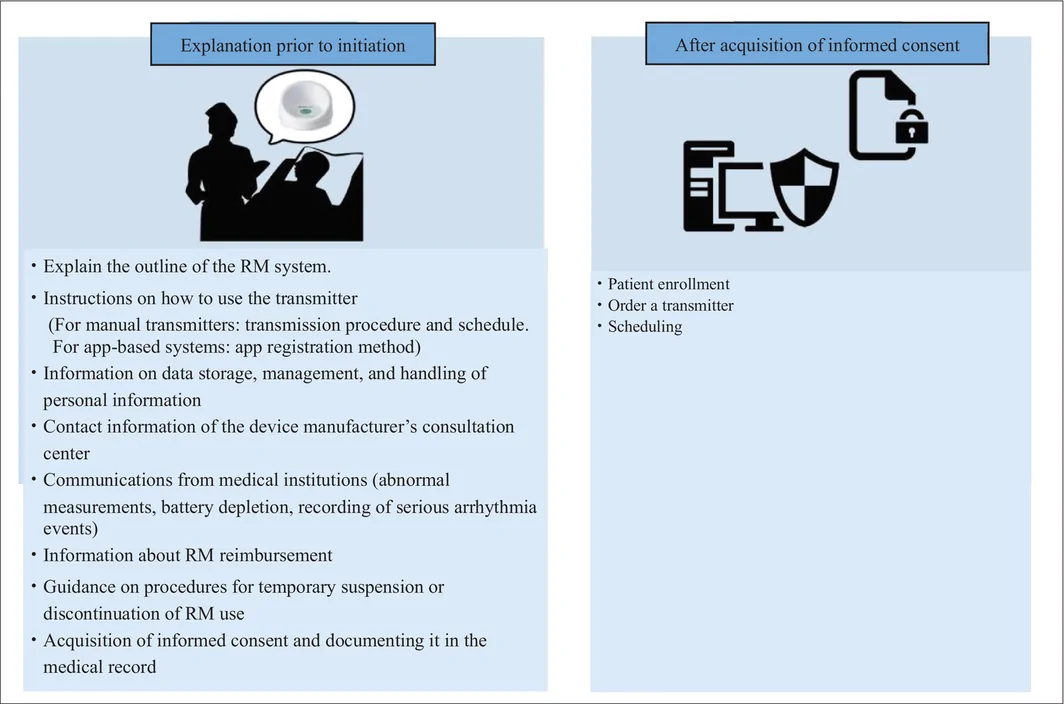
Work needed when introducing a remote monitoring system and after obtaining informed consent. This schematic illustrates the workflow required for implementation of a remote monitoring (RM) system, including patient education prior to initiation and operational procedures after informed consent. Key steps include explanation of system functions, transmitter setup, patient enrollment, and scheduling of RM data transmission.

The introduction of RM reduces the number of outpatient visits and extends the interval between visits, thereby decreasing the need for in‐person consultations and lessening the burden on patients and their families of visiting the hospital [7, 9, 45]. In addition, the introduction of a RM enables the early detection of arrhythmia events and abnormal measurements that could otherwise only be identified through conventional in‐person device checks [43]. This can be expected to shorten hospital stays and improve clinical outcomes [5, 10, 46]. In addition to explaining these clinical benefits to patients, refer to the need for patients' cooperation in using the RM (in terms of the places available to place repeaters to maintain signal status and keeping them plugged in). Also, patients and their families should be made aware that RM is not designed to serve as an emergency response system (Figure 4).

### 4.3 Use of Remote Monitoring Transmitter

**FIGURE 5:**
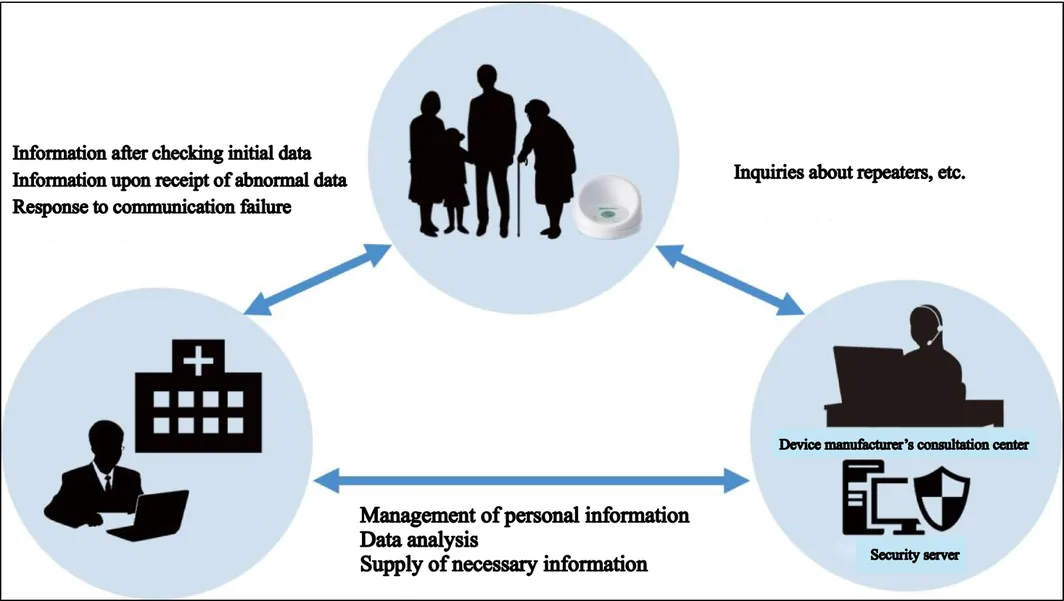
Actions to be taken after introduction of remote monitoring system. This schematic illustrates the interaction among patients, healthcare providers, and device manufacturers after RM initiation. Data transmitted from cardiovascular implantable electronic devices (CIEDs) are analyzed at the medical institution, while manufacturers maintain secure data servers and provide technical support to ensure stable system operation.

Provide instructions on the type of transmitter (fixed, portable, or smartphone app‐based) and how to operate it and instruct patients to maintain an environment that allows stable communication regardless of the transmitter type [43]. Once initial data transmission is confirmed, the patient should be informed of this. If installation of the transmitter is delayed or if communication remains unsuccessful, the situation should be promptly investigated and corrective steps taken to ensure stable transmission (Figure 5).

In the case of a manually operated transmitter, it is important that patients or their families know how to correctly operate the transmitter. Instruct patients or their families and obtain their cooperation to send data according to the schedule provided by their medical institution. If problems arise, patients should be directed to contact the device manufacturer's consultation center as specified in the instruction manual. In cases where transmitter configuration changes after a model upgrade or following device replacement due to battery depletion, patients and families should be informed accordingly. Patient education on the use of transmitters should be individualized and reinforced on an ongoing basis according to the level of understanding of patients and their families.

### 4.4 Data Saving, Management, and Viewing on a Security Server Located Outside Japan

In addition to explaining the RM and the frequency of data transmission. Patients and their families should be informed that all personal and medical information obtained through the system is securely stored on the device manufacturer's server, which is located outside the country, in accordance with international standards for data protection and confidentiality. Access to this data is strictly limited to authorized medical professionals who connect via a secure Internet system using individual IDs and passwords. Providing this information helps enhance patient confidence in data security and privacy protection. When the managing medical institution changes due to patient relocation or transfer, the referral document should clearly state that the patient is enrolled in a RM program. Both the transferring and receiving institutions should explain to patients or their families that the data transfer and management are carried out in accordance with the agreements and workflow of each medical institution.

### 4.5 Visiting a Medical Institution When Contacted or When Symptoms Develop

Each institution should establish an internal workflow to be followed when problems such as abnormal measurements, battery depletion, serious arrhythmias, or other findings requiring early intervention are identified [43]. Patients should be informed that medical professionals may contact them according to each institution's operational procedures. In addition, they should be advised that the data obtained through the RM system are not continuously reviewed, and that the system cannot evaluate conditions beyond its measurable parameters—such as the patient's symptoms or overall clinical status. Patients should therefore be instructed to visit a medical institution promptly if symptoms develop, to ensure timely and appropriate management.

### 4.6 Reimbursement for Remote Monitoring

Explain to patients that an additional reimbursement for RM will be claimed when they visit the device outpatient clinic. When the service is provided under the national health insurance system, the patient's copayment amount may vary depending on factors such as the certified grade of physical disability, individual copayment rate, and any revisions in the reimbursement points designated for RM (Table 7).

**TABLE 7:** Examples of medical fees claims for remote monitoring services as of August 2024.

Month	1	2	3	4	5	6	7	8	9	10	11	12
Pacemaker	260 points (reimbursement for RM)	260	260	260	260	260	260	260	260	260	260	300 Guidance and management fee
ICD/CRT‐D	480 points (reimbursement for RM)	480	480	480	480	520 Guidance and management fee	480 RM (reimbursement)	480	480	480	480	520 Guidance and management fee

*Note:* Medical fee examples are based on the Japanese national health insurance reimbursement system as of August 2024. Actual claims may vary according to institutional policies and insurance revisions.

In the case of patients with pacemakers, the RM fee can be claimed once per month. For example, when an outpatient visit is made after 12 months, the management fee and 11 months of RM fees are billed as follows: 260 (points) × 11 months + 300 (points) = 3160 (points). For patients with an ICD or CRT‐D, the reimbursement rate for RM is higher than that for pacemakers. When outpatient visits are made twice a year (subject to management fee) and 10 months of the RM services are provided, the total claimable amount is calculated as: 480 (points) × 10 months + 520 (points) × 2 months = 5840 (points).

### 4.7 Interruption or Discontinuation of the Remote Monitoring

Patients should be instructed to keep their contact information up to date and inform their medical institution in advance if they are going to be away for an extended period due to hospitalization or travel. They should also be informed to contact their medical institution if they wish to discontinue the use of RM or in the event of the patient's death. When discontinuation is decided, the patient (or family) should be informed of the procedure for returning or disposing of the transmitter and deleting the smartphone application. The medical institution should promptly register the discontinuation in the system.

### 4.8 Obtaining and Withdrawing Consent

When introducing an RM system, patients and their families should be given a written explanation and informed consent must be obtained. Whether consent was obtained or not must be recorded in the patient's medical record, and the signed consent form must be retained in the patient's medical record [43]. In addition, patients should also be informed that consent can be withdrawn at any time, and that they should notify the medical institution if this decision is made. Upon receiving a request for withdrawal of consent from the patient or family, the medical institution should respond promptly and appropriately.

### 4.9 Registration and Handling of Personal Information

Patients who use an RM system must be registered. The purpose of using personal information related to RM should be clearly explained (Table 8). Patients and their families should also be informed that the personal information of related patients (including details regarding CIEDs) is protected by the Personal Information Protection Act. They should be advised that such information may be shared only with authorized medical professionals and employees of the device manufacturer and will not be disclosed to any third parties or used beyond its intended purpose of use [47]. In addition, it should be explained that the patient's personal information will be transferred to a server operated by a company affiliated with the CIED manufacturer, and that strict management procedures are implemented to ensure data protection in accordance with the eight OECD Privacy Guideline Principles [48, 49].

**TABLE 8:** Objectives of using personal information related to the remote monitoring system.

Send the transmitter to the patient. Assign the transmitter to the patient's CIED. Maintain the remote monitoring system. Provide technical and safety information related to the products of the device manufacturer to the responsible medical professionals. Provide information upon request from the responsible medical professionals. Prevent health hazards related to the products of the device manufacturer, or take appropriate measures in the event of such incidents. Provide information for surveys or product development concerning the performance, usability, efficacy, safety, and dissemination of the manufacturer's products. Provide information for epidemiological or academic research, as well as for presentations or publications of such research at academic conferences. Respond to requests from medical professionals at medical institutions for information for research purposes; however, such data must be processed to prevent identification of individual patients.

*Note:* Personal information management is conducted in accordance with the Act on the Protection of Personal Information in Japan and international privacy standards including OECD privacy guidelines.

### 4.10 Synopsis

When introducing an RM system, patients and their families should be informed of the benefits of RM (fewer outpatient visits, early detection of arrhythmias, improved prognosis, etc.) and the desirability of introducing it as early as possible. Patient education should include the types of transmitters, their operation, and the importance of maintaining stable communication. Patients should be notified once the first data transmission is confirmed. Patients should be advised that the medical institution may contact patients depending on the importance of an alert and that it is important for them to seek medical attention at their own discretion if symptoms appear. Make efforts to gain patients' confidence by informing them that their data are stored securely on servers outside the country and can only be accessed by a limited number of authorized medical professionals. Patients should also be informed that a reimbursement fee for RM will be claimed and that they should notify the medical institution in advance of any prolonged absence such as travel or hospitalization. The procedures for discontinuing RM, as well as for obtaining and withdrawing consent, should be clearly explained. In addition, patients and their families should be educated about the protection and management of personal information to ensure their understanding and trust. These efforts will enable patients and their families to use RM with confidence. The evidence levels for patient education regarding the introduction of RM systems are shown in Table 9 [43]. Although certain operational aspects described in this statement reflect characteristics of the Japanese healthcare system—such as reimbursement structures and clerical roles—most principles presented here, including multidisciplinary team management, alert‐based monitoring strategies, and patient education, are broadly applicable to remote monitoring programs internationally.

**TABLE 9:** Patient education's evidence levels for introducing the remote monitoring system [43].

Recommendation class	Evidence level	Recommendation
I	C‐LD	Conduct patient's education on the RM system continually considering the degree of understanding by patients, their families and caregivers [50, 51].
I	C‐EO	Explain that consent to RM can be withdrawn or RM can be stopped at the request of patients or their families.
IIa	C‐EO	Consider providing practical education and guidance using the transmitter [52].
IIa	C‐EO	Consider providing explanations on the claim of RM premium associated with the use of the RM system [52].
IIa	C‐EO	Consider providing explanations to patients that they may be given an alert from the medical institution depending on its importance but the RM system is not intended to be an emergency response system.
IIa	C‐EO	Consider providing explanations that personal information and data from the RM system will be saved on a device manufacturer's secure server located outside Japan and will not be divulged to any unauthorized person or used outside its intended scope of use.

*Note:* Recommendations are based on evidence from studies evaluating patient education and acceptance of remote monitoring systems and on expert consensus regarding clinical practice. Colour‐coded in descending order of recommendation strength (green, yellow, pale yellow, orange) and evidence level (dark blue gradually lightening to pale blue).

## 5 Roles and Training of Remote Monitoring Management Team Members From a Variety of Professions

### 5.1 Introduction

As defined in the Introduction of this statement, RM encompasses both remote interrogation (RI), which refers to scheduled or patient‐initiated device transmissions, and remote monitoring (RM), which refers to automatic alert‐based transmissions. RM of CIED patients should be managed by a multidisciplinary team comprising physicians and a range of allied medical professionals. A typical RM team includes physicians, nurses, clinical engineers, clinical laboratory technicians, heart failure care educators, radiology technologists, and administrative staff (clinical clerks).

**FIGURE 6:**
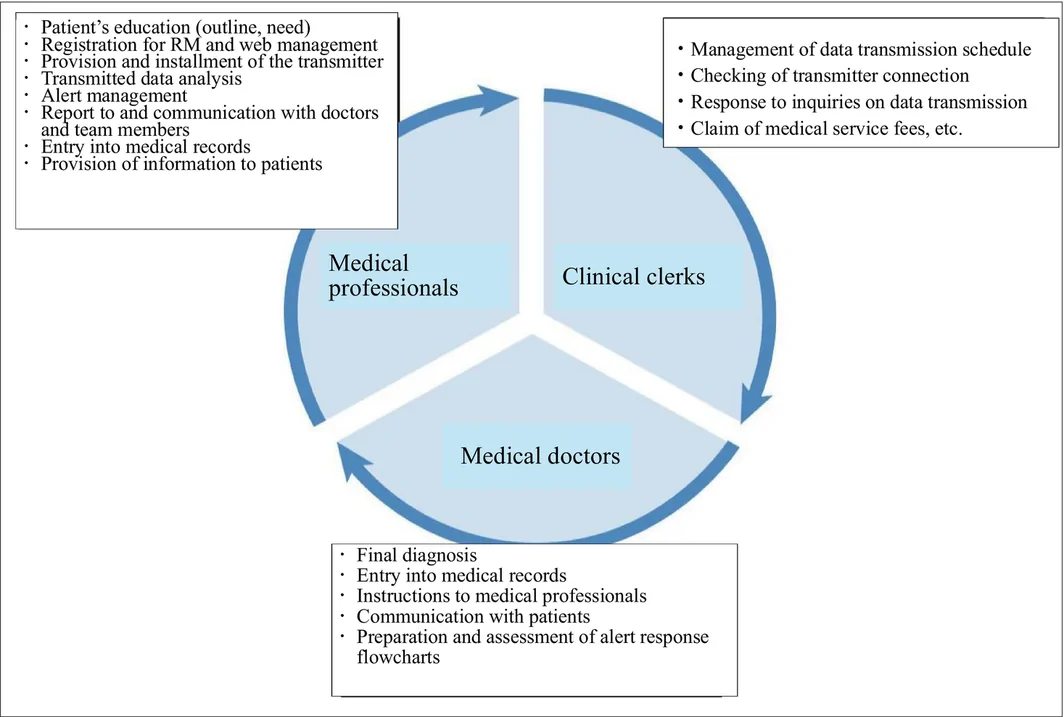
Roles of remote monitoring management team members from different professions. This diagram illustrates the roles of healthcare professionals involved in RM management. Physicians provide clinical oversight and final decision‐making, whereas allied professionals conduct data analysis, alert management, and patient education. Administrative staff support operational tasks such as scheduling and documentation.

Each team member should be assigned clearly defined roles and responsibilities, with regular information sharing and coordination to ensure effective operation (Figure 6) [53]. Building upon the 2015 HRS Consensus Statement, RM management has continued to evolve [54]. With the growing number of patients enrolled in RM programs, the expanding use of ILRs, and the increasing sophistication of alert response workflows, the structure and staffing of RM management teams warrant reassessment. Consensus Statement highlights the rising workload associated with frequent data transmissions and emphasizes the need for adequately trained support staff, detailing their specific roles within the RM management framework [43].

### 5.2 Roles and Responsibilities of Physicians

Physicians hold overall responsibility for RM management. Data transmitted via RM and the analyses performed by other medical professionals—such as nurses and clinical engineers—are ultimately reviewed and interpreted by physicians, who make clinical judgments and determine appropriate treatment strategies based on these findings. Furthermore, the design and implementation of RM‐based treatment protocols and workflows are primarily developed under the direction of physicians at each institution, with final decisions made under their authority. For this reason, doctors responsible for RM management must understand all CIED data. Accordingly, physicians overseeing RM must possess a comprehensive understanding of all CIED‐related data and intracardiac electrograms, as well as the expertise required to lead troubleshooting efforts and manage heart failure care. Therefore, it is recommended that physicians responsible for supervising and managing RM obtain certification as JHRS‐certified arrhythmia specialists [53].

### 5.3 Roles and Responsibilities of Medical Professionals

Medical professionals such as nurses and clinical engineers play a central role in the practical management of RM for patients with CIEDs. Their responsibilities include providing patients with initial guidance and education on the outline of RM, management of personal information, installation and use of transmitters, and procedures for online patient registration. They are also responsible for scheduling, analyzing transmitted alert data, and initiating appropriate patient interventions when necessary [43]. In addition, medical professionals should establish standardized policies and procedures for managing RM schedules and responding to emergency alerts. In consultation with the supervising physician, they should define in advance: (1) protocols for using RM data as a substitute for in‐person programmer checks; (2) methods and procedures for alert management (including standardization of alert types and reference thresholds); (3) standard operating procedures (SOPs) and score sheets; (4) working‐hour systems (e.g., whether 24‐h coverage is available); (5) data review schedules; and (6) procedures for reporting findings to the physician responsible for RM oversight. It is also important to establish a system that allows appropriate contact with patients depending on the situation. To minimize data oversights and misjudgments, a designated person should be assigned responsibility for alert management, with clearly defined duties. When an alert or other notable event occurs, key information should be documented in the patient's medical record, and the relevant RM data (e.g., PDF output) should be attached. In the absence of significant findings, a monthly record should state that “device function was evaluated via RM and no clinically significant changes were observed,” with confirmation by the attending physician to ensure continuity of RM documentation. It is recommended that medical professionals involved in RM are encouraged to obtain certification as a JHRS‐certified CIED Medical Professional or Cardiac Device Representative (CDR) [55]. Furthermore, incorporating a Japanese Circulation Society (JCS)‐certified Heart Failure Educator into the RM management team may further enhance the quality and comprehensiveness of patient care.

### 5.4 Roles and Responsibilities of Clinical Clerks

Although RM enhances patient satisfaction, it puts an increased burden on physicians and other medical professionals. Clinical clerks can play an important role in alleviating this burden by assuming responsibility for non‐clinical tasks associated with RM. According to the 2023 HRS Consensus Statement, such responsibilities include assisting with patient registration, contacting patients to request data transmission, scheduling transmission sessions, and verifying transmitter connection status [43]. They should also register RM data files (e.g., PDFs) in the electronic medical records and assist in documenting related information on behalf of clinical staff. It is especially important to check connection status and respond promptly to delays in transmitter installation and transmission interruptions to ensure stable communication. Delegating these operational and administrative duties to clinical clerks can substantially reduce the workload of physicians and other medical professionals. In order to carry out these tasks properly, however, it is essential to clarify the scope of these tasks and develop detailed operational manuals and training protocols.

### 5.5 Synopsis

Managing RM smoothly and accurately requires a multidisciplinary team centered around doctors that includes nurses, clinical engineers, clinical laboratory technicians, heart failure educators, and clinical clerks. Smooth and accurate management of RM requires a multidisciplinary team led by physicians and is composed of nurses, clinical engineers, clinical laboratory technicians, heart failure educators, and clinical clerks. The roles and responsibilities of each team member should be clearly defined, with an established management workflow and shared regular information sharing to ensure coordinated operation. The respective roles of RM and the corresponding levels of evidence supporting their education and training are summarized in Table 10 [56].

**TABLE 10:** Roles of each profession included in the remote monitoring management team and evidence levels for their education [56].

Recommendation classes	Evidence level	Recommendation
I	C‐EO	Organize a multidisciplinary team around a medical doctor to ensure that RM patient management is conducted smoothly and accurately Establish a physician‐led multidisciplinary team to ensure smooth and accurate RM management [43, 56].
I	B‐NR	Prepare a workflow for each profession to clarify its role and responsibility in RM [56, 57].
IIa	B‐NR	Take sufficient time to execute RM‐related activities smoothly Sufficient time should be allocated to ensure that RM‐related activities are carried out smoothly [58].

*Note:* RM management requires a multidisciplinary team including physicians, nurses, clinical engineers, technicians, and administrative staff. Roles may vary depending on institutional workflow and staffing resources. Colour‐coded in descending order of recommendation strength (green, yellow, pale yellow, orange) and evidence level (dark blue gradually lightening to pale blue).

## 6 Management of Remote Monitoring Data

### 6.1 Frequency of Remote Monitoring Data Transmission

As defined above, RM in this document includes both remote interrogation (RI) and automatic remote monitoring functions depending on device capabilities. Remote monitoring functions of implantable devices vary by device type and manufacturer. Some systems automatically transmit data in real time or on a daily basis without patient intervention, while others require manual operation to send data. For patients with pacemakers or ICDs, data should be collected every 3 months (four times a year) [59]. In patients with heart failure or those with ILRs, data should be collected monthly. Even when RM is performed continuously, at least one in‐person consultation per year is recommended. This visit allows healthcare providers to claim reimbursement for RM and to update medical records with any events or medication changes that have occurred since the previous visit. For devices without an automatic capture algorithm, the in‐person consultation also provides an opportunity to assess the pacing threshold, verify the function of automatic sensing and capture algorithms, and adjust parameters as necessary. It also provides an important opportunity to address any questions or concerns patients may have.

### 6.2 Alert Settings and Follow‐Up System

**FIGURE 7:**
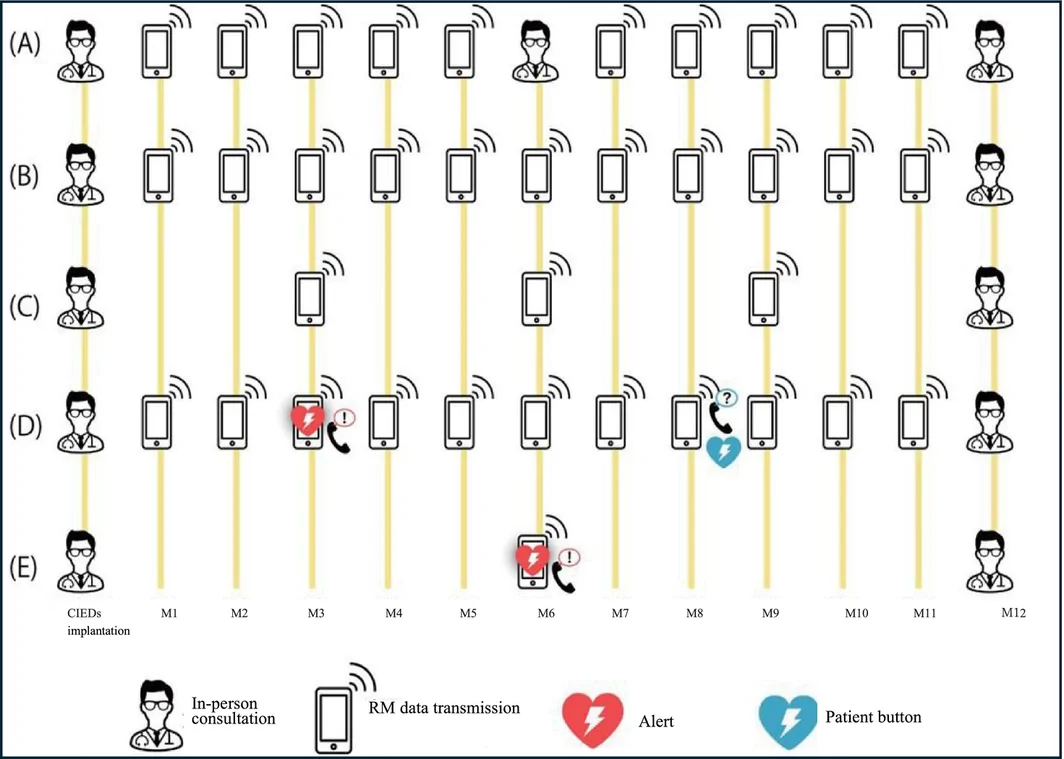
Examples of patient management by remote monitoring. In case RM is started after CIED implantation—(A) In‐person consultation twice a year while continuing RM every month (in persons with implanted pacemakers for instance); (B) In‐person consultations once a year while continuing RM every month (in persons requiring ILR or heart failure management for instance); (C) In‐person consultations once a year with RM 3 times a year; (D) The medical institution may contact the patient when the alert is activated or the patient may contact the medical institution by pushing the patient's button on the management device; (E) Alert‐based monitoring with an in‐person consultation once a year and response only when the alert is activated (in stable persons with an implanted pacemaker for instance).

In addition to the periodic transmission described above, alerts are generated according to settings configured by medical professionals and the specific transmission and reception interval defined by each device manufacturer. At a minimum, alerts should be set to battery status, lead integrity, and arrhythmia events. Several factors can hinder the collection and transmission of device and patient information. Examples include technical errors in transmitter setup, communication failures between the CIEDs and transmitter, and alerts not being delivered to the healthcare professional. Therefore, a system must be established to ensure that patients remain connected to the monitoring platform, that data are transmitted at the designated intervals, and that relevant findings are promptly communicated to both patients and medical professionals. In the past, regular in‐person consultation was the primary method of follow‐up, with RM positioned as a complementary tool [60]. However, a US study found that this resulted in an in‐person consultation rate of less than 50% [61]. As increasing evidence supporting the usefulness and superiority of RM, the 2015 HRS Expert Consensus explicitly recommended a system in which in‐person consultations are basically limited to once a year, RM is performed periodically, and additional responses are made to ad hoc alerts triggered by RM [54]. The details and recommended frequency of CIED monitoring are shown on Figure 7. The benefits of RM are generally greater in patients with ICDs or CRT‐Ds than for those with pacemakers. Nevertheless, adopting a uniform follow‐up protocol for all patients with CIEDs would offer advantages, including reduced workflow complexity, simplified management plans, and improved protocol adherence. According to the 2023 HRS Expert Consensus, patients with pacemakers or ICDs who do not have heart failure and can maintain stable daily RM data transmission may be managed using an alert‐based RM approach without routine 3‐month checks. In such cases, outpatient visits may be reduced to once every 2 years; however, in Japan, one in‐person visit per year remains necessary to meet reimbursement requirements. Patients should be informed that while RM alerts will not be subject to an emergency response, any received alerts will be investigated and dealt with within 2 days. Alert‐based CIED management is expected to improve adherence and engagement among both patients and medical professionals.

### 6.3 Recording of Remote Monitoring Reports in Medical Records

The recommended data collection interval is four times a year for patients with pacemakers or ICDs and once per month for patients with heart failure, and reports should be prepared in accordance with these intervals. However, the review, interpretation, and recording of RM data should follow the workflow established at each institution. Information obtained through RM is important for information sharing among patients, referring physicians, and heart failure specialists.

### 6.4 Use of Third‐Party Resources for Remote Monitoring Management

**FIGURE 8:**
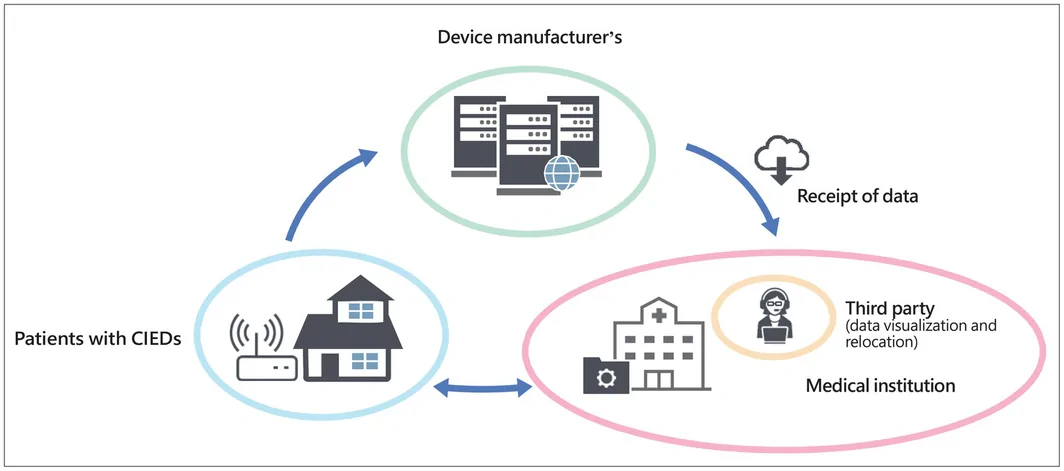
Remote monitoring involving third parties (example). This schematic illustrates an RM architecture incorporating third‐party data management systems. Device data transmitted from patients with CIEDs are stored on manufacturer‐managed servers and subsequently integrated into hospital electronic medical records through data‐visualization platforms, enabling unified data management across device manufacturers.

As the number of remotely monitored patients increases, it will be difficult to continue managing them properly without the support of specialized staff and other relevant personnel. Many facilities face challenges in establishing the necessary infrastructure for RM due to recent changes in labor laws and restrictions on overtime work. In Japan, employees of device manufacturers are not permitted to perform RM or provide patient care on behalf of medical professionals. However, systems have become commercially available that allow medical professionals to log in to RM websites provided by multiple device manufacturers and import patient data directly into their electronic medical records (Figure 8). Because each manufacturer maintains its own unique RM system, operational management has traditionally required accessing each company's website each time. Under the leadership of the HRS, unified data specifications—known as *implantable device cardiac observation profiles*—have been developed to standardize data collection from pacemakers, ICDs, and CRT‐Ds across manufacturers [62, 63]. These specifications enable seamless data management and transfer to electronic medical records regardless of device type. RM management system software developed in Japan allows data from multiple manufacturers to be viewed on a single screen, allowing for sorting, searching, and utilization of alert information, as well as entry and transfer to electronic medical records. In addition, some software supports accounting procedures through integration with the medical fee claims system (Table 11). Because RM in Japan is reimbursed under the national insurance system, hospitals can generate revenue even after accounting for software usage fees. The adoption of these integrated systems enables more effective and efficient RM operations for both patients and medical professionals.

**TABLE 11:** Comparison of unified CIED remote monitoring management systems (as of June 2025).

	CardioAgent Pro for CIEDs	ORFICE	REVOLVER
Incorporated major device manufacturers	Abbott, Biotronik, Boston Scientific, Medtronic, MicroPort	Abbott, Biotronik, Boston Scientific, Medtronic, MicroPort	Abbott, Biotronik, Boston Scientific, Medtronic
Collective display of API data	〇	〇	〇
Additional registration of collateral information	〇	〇	〇
PDF registration in electronic medical charts and report drafting	〇	〇	〇
Linkage using patients' ID card Nos., etc.	〇	〇	〇
Linkage of appointments for outpatient consultation	〇	〇	〇
Handling of medical bills		〇	

*Note:* Systems listed are examples of currently available software platforms for integrating RM data from multiple device manufacturers. 〇 indicates it is compatible.

Abbreviation: API, application programming interface.

### 6.5 Possibility of Multicenter Big Data Collection and Analysis

Through RM systems, large amounts of physiological information are collected from CIEDs via websites. Manufacturers are responsible for using these data to improve their product performance and safety continuously [64]. At the same time, such data hold significant potential for research aimed at detecting early disease onset developing newer therapeutic strategies and identifying previously unrecognized pathophysiological mechanisms.

### 6.6 Synopsis

In RM management, data should be collected every 3 months from patients with pacemakers or ICDs, and monthly from patients with heart failure and patients with ILRs. At least one in‐person consultation per year is recommended. Information obtained from RM is essential for sharing information among patients, referring physicians, and heart failure specialists. Recent evidence indicates that in patients with stable pacemakers or ICDs capable of continuous daily data transmission, in‐person consultation intervals can be safely extended to 24 months. Using software products manufactured by third parties, data from multiple device manufacturers can be centrally managed and incorporated into electronic medical records. These systems enhance patient privacy protection while reducing staff workload. RM systems also facilitate the collection and analysis of multicenter big data, which can be leveraged for product improvement, early disease detection, and clinical research. Recommendations regarding RM implementation and in‐person consultations are summarized in Table 12, while Table 13 presents recommendations regarding the alert timeline, and Table 14 provides guidance on incorporating RM reports into electronic medical record using third‐party data management software.

**TABLE 12:** Recommendations regarding remote monitoring implementation and in‐person consultations.

Recommendation classes	Evidence level	Recommendation
I	A	Conduct in‐person consultations at least once a year and perform device interrogations using a programmer even if patients are managed by RM [42].
I	B‐R	Implement continuous daily remote monitoring in CIED patients with device recalls or safety advisories to allow early event detection [4, 5, 14, 20, 41, 61, 65, 66, 67].
IIa	B‐R	Consider extending the in‐person consultation interval to up to every 24 months in stable pacemaker patients connected to RM capable of continuous daily data transmission [8, 9, 68] (For the sake of health insurance coverage, in‐person consultations are recommended once a year).
IIa	B‐R	Consider extending the in‐person consultation interval to up to every 24 months in stable ICD patients connected to RM capable of continuous daily data transmission [5, 14, 45, 69, 70] (For the sake of health insurance coverage, in‐person consultation is recommended once a year).

*Note:* Recommendations are based on published randomized trials and international consensus documents regarding CIED follow‐up strategies. Annual in‐person visits are recommended in Japan primarily due to reimbursement requirements. Colour‐coded in descending order of recommendation strength (green, yellow, pale yellow, orange) and evidence level (dark blue gradually lightening to pale blue).

**TABLE 13:** Recommendations regarding the alert timeline.

Recommendation classes	Evidence level	Recommendation
I	C‐EO	Inform patients with CIED managed by RM, their families or caregivers that automatic alerts transmitted through RM cannot serve as a substitute for an emergency management system.
IIa	C‐EO	Review high‐priority alerts and take appropriate action within 2 days, excluding non‐consultation days.

*Note:* Alert response timelines should be determined according to institutional protocols. RM alerts should not be considered an emergency response system. Colour‐coded in descending order of recommendation strength (green, yellow, pale yellow, orange) and evidence level (dark blue gradually lightening to pale blue).

**TABLE 14:** Recommendations regarding the incorporation of remote monitoring reports into electronic medical charts using data management software manufactured by third parties.

Recommendation classes	Evidence level	Recommendation
I	C‐EO	Ensure patient privacy is protected when incorporating RM reports into electronic medical records using third‐party data management software.
IIa	C‐EO	Consider using third‐party data management software to reduce RM‐related burdens of workload [58].
IIa	C‐EO	Consider informing CIED‐implanted patients that third‐party data management software will be used to enhance the quality of their care.

*Note:* Data integration using third‐party software should comply with institutional privacy policies and national regulations on medical information security. Colour‐coded in descending order of recommendation strength (green, yellow, pale yellow, orange) and evidence level (dark blue gradually lightening to pale blue).

## 7 Responsibilities and Required Actions of Device Manufacturers

### 7.1 Actions at the Time of Implantation (Including Initial Setup) and in the Event of a Recall

The responsibilities of device manufacturers include ensuring the safety and effectiveness of their products, in addition to developing reliable RM management technologies. To support these efforts, continuous data collection and evidence building are essential, along with the creation of technologies that can be effectively deployed in future clinical practice. CIED functions and clinical data obtained from RM are stored on servers owned and managed by device manufacturers, where a vast amount of user information is accumulated. Therefore, the highly secure management of patients' personal information is extremely important. The data stored on device manufacturers' servers have significant value for quality assurance, technological advancement, and program improvement (e.g., tracking device performance and detecting early signs of potential device malfunctions that require attention). Because these data can play a key role in addressing inquiries from medical professionals and researchers, device manufacturers should establish an independent department dedicated to facilitating scientific review and appropriately responding to research‐related requests [43].

### 7.2 Continuity of Remote Monitoring

While RM benefits patients by providing early notification of critical events, continuous connectivity is essential to achieve these advantages. To ensure sustained effectiveness, it is essential to involve all stakeholders—patients, medical professionals, and industry partners—in the development and design of RM systems. Such collaboration helps adapt RM technologies to evolving digital environments and enhances convenience, flexibility, and reliability. Device manufacturers should offer alternative systems for patients with limited mobile network access or low digital literacy and ensure that system updates do not impose a financial burden on users. To promote continued utilization, RM applications must remain compatible with commonly used smartphones and be regularly updated to maintain functionality and security [58]. Furthermore, device manufacturers should develop technology that notifies patients of transmission status and prompts them to reconnect if communication is interrupted [71, 72].

### 7.3 Response to Recalls and Safety Recommendations

In the event of a recall or safety recommendation, device manufacturers must promptly notify both medical institutions and affected patients. Internationally, close collaboration among the device industry, the government (U.S. Food and Drug Administration), and scientific societies ensure that device manufacturers promptly notify doctors and patients in case of recalls or safety advisories occur [73, 74]. In Japan, in the event of a product recall, device manufacturers promptly report to the relevant prefectural government. The recall class is then determined, and countermeasures are considered accordingly. Device manufacturers are required to promptly report the recall decision to the concerned medical institutions or patients (ideally within 24 h). Notifications to medical device safety control managers and medical professionals are delivered by fax, email, or verbal communication, and manufacturers must obtain a signed acknowledgment confirming receipt of the report. If patients learn of recall through the media before being informed by their medical institution, this may increase anxiety and dissatisfaction. Therefore, notifying patients immediately is essential for medical institutions [75]. To facilitate rapid and broad dissemination of information, device manufacturers are also required to publish all recall information on their official pharmaceutical and medical device information websites and to issue press releases to the media [43].

### 7.4 Attendance to Ensure Proper Use of Medical Devices

**FIGURE 9:**
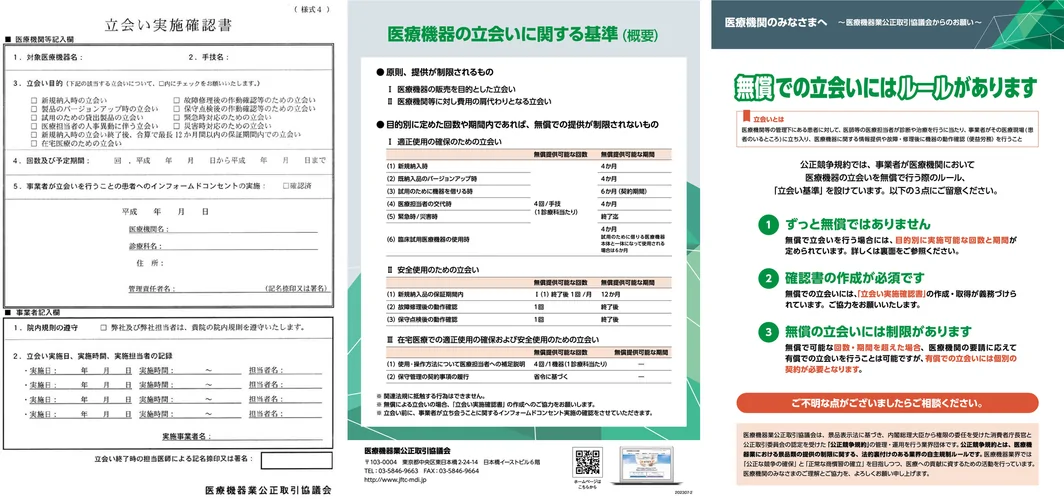
Attendance of medical device manufacturers. This figure shows representative documents used to confirm manufacturer attendance for ensuring appropriate use of medical devices. Such documentation supports regulatory compliance, transparency, and appropriate technical assistance during device‐related procedures or training activities.

There are currently restrictions on attendance to ensure the proper use of newly delivered medical devices due to concerns about conflicts of interest and personal information protection (Figure 9). For initial introduction, up to four complimentary attendances per department are permitted within a 4‐month period, provided that written confirmation of attendance is obtained. Thereafter, attendance is permitted only under a fee‐based contract between the medical institution and device manufacturer employing a certified CDR.

### 7.5 Provision of Support Services to Medical Professionals

Device manufacturers play an important role in providing education and training to medical professionals involved in RM. There are many differences between manufacturers in the programming of RM systems and devices. Therefore, providing guidance to RM managers regarding unique alerts and parameters for various clinical events can minimize inappropriate alerts and the need for reprogramming. Involving patients and medical professionals (who have no conflicts of interest with the device manufacturer) in technology development can enhance the reliability, efficiency, and user satisfaction of RM.

### 7.6 Synopsis

Device manufacturers are responsible for developing RM technology, ensuring its safety and effectiveness, and contributing to the data collection and building evidence. They must provide timely information and respond promptly in the event of a recall or safety advisory. Manufacturers should also provide alternative systems for patients with limited mobile connectivity or low digital literacy to ensure equitable access and minimize financial burden. Device manufacturers play an important role in educating and training medical professionals involved in RM, thereby enhancing system reliability and user satisfaction. Recommendations regarding the role of device manufacturers in optimizing individual patient care are summarized in Table 15 [43]. Recommendations regarding the management of RM recalls and safety advisories are presented in Table 16, and recommendations regarding support provided before and after implantation are shown in Table 17 [43].

**TABLE 15:** Recommendations on the role of device manufacturers in optimizing individual patient care [43].

Recommendation classes	Evidence level	Recommendation
I	C‐EO	Device manufacturers should provide appropriate education and technical support to healthcare professionals and ensure effective communications with individual patients.
I	C‐EO	Device manufacturers should provide reliable, safe, and accurate RM services that ensure patient satisfaction.
I	C‐EO	Device manufacturers should actively involve patients and healthcare professionals in the technical design and development of RM.
I	C‐EO	When RM communications are interrupted, device manufacturers should promptly notify medical institutions and take appropriate action to restore connectivity.

*Note:* Device manufacturers are responsible for ensuring the safety, reliability, and technical support of RM systems while collaborating with healthcare professionals and regulatory authorities. Colour‐coded in descending order of recommendation strength (green, yellow, pale yellow, orange) and evidence level (dark blue gradually lightening to pale blue).

**TABLE 16:** Recommendations on the role of device manufacturers in managing remote monitoring recalls and safety advisories.

Recommendation classes	Evidence level	Recommendation
I	C‐EO	Device manufacturers should immediately notify medical institutions in the event of a recall or safety advisory.

*Note:* In the event of a recall or safety advisory, manufacturers should notify medical institutions promptly in accordance with regulatory requirements. Colour‐coded in descending order of recommendation strength (green, yellow, pale yellow, orange) and evidence level (dark blue gradually lightening to pale blue).

**TABLE 17:** Recommendations on support provided by device manufacturers before and after implantation [43].

Recommendation classes	Evidence level	Recommendation
I	C‐EO	Device manufacturers should encourage healthcare professionals to implement RM procedures within 2 weeks before or after implantation.
I	C‐EO	Device manufacturers should provide appropriate methods for configuring alert settings to healthcare professionals.

*Note:* Education and technical support provided by manufacturers should follow institutional policies and conflict‐of‐interest regulations. Colour‐coded in descending order of recommendation strength (green, yellow, pale yellow, orange) and evidence level (dark blue gradually lightening to pale blue).

## 8 Usefulness and Optimization of Alert Setting and Alert‐Based Monitoring

As discussed above, many benefits of RM have been reported, with one of the best‐established benefits being its ability to reduce the number of outpatient visits without compromising safety while enabling early detection of clinically significant events [43, 76]. This benefit has been confirmed in a prospective clinical study of pacemaker‐implanted patients conducted in Japan as well [9]. However, much of this evidence is obtained from RM with continuous device monitoring and automated alert functions. Because routine review of RM data can impose a substantial workload on medical staff, the concept of alert‐based monitoring—responding only when alerts are triggered—has gained wide acceptance. The usefulness of this alert‐based monitoring approach has been demonstrated primarily in the TRUST trial and its substudies [4, 66, 70]. When setting alert parameters, it is necessary to aim for settings that enable early detection of abnormalities that would otherwise be identified during in‐person device follow‐ups, while ensuring that the alerts are clinically actionable and minimizing unnecessary notifications.

### 8.1 Recommended Alert Setting Parameters for Pacemakers and ICDs/CRTs

Some devices—such as certain leadless pacemakers—lack RM functions and therefore do not have alert settings (Table 3). Recommended alert settings for other types of CIEDs are summarized in Table 18. In this classification, red alerts indicate conditions that require an emergency or semi‐emergency response, whereas yellow alerts indicate events that warrant an early but non‐urgent response (Table 19).

**TABLE 18:** Recommended alert settings for CIEDs.

Recommendation classes	Evidence level	Recommendation
I	B‐R	For all patients under remote monitoring (RM), and particularly for those with implanted devices or leads subject to a recall or safety advisory, alert settings are recommended for device reset, battery depletion or end of service, lead polarity switch, and abnormal electrical parameters such as out‐of‐range pacing thresholds and lead impedance values [77, 78, 79, 80, 81].
I	C‐LD	Set alerts for all shock and ATP therapies in patients implanted with ICD [15, 70, 82, 83].
II a	B‐R	In patients with implanted devices (in particular ICD/CRT‐D) that can monitor heart failure markers, consider reporting the monitoring results to patients and taking proactive measures to prevent worsening of heart failure [35, 38, 83, 84, 85].
II a	C‐LD	Consider setting an alert for patients with implanted CRT devices when the biventricular pacing rate falls below the specified threshold [83, 86].
II a	C‐LD	Consider setting an alert when the atrial high‐rate episode (AHRE) duration or rate exceeds the predefined threshold [15, 87, 88, 89].
II a	C‐EO	Consider setting an alert for a high right ventricular pacing rate in patients with pacemakers implanted for sick sinus syndrome or in CIED‐implanted patients with reduced left ventricular function [90].

*Note:* Alert parameters should be tailored to patient condition, device type, and clinical indication. Recommended settings are based on international guidelines and prior RM studies. Colour‐coded in descending order of recommendation strength (green, yellow, pale yellow, orange) and evidence level (dark blue gradually lightening to pale blue).

**TABLE 19:** Recommendations of red alert and yellow alert settings for pacemakers and ICDs/CRTs.

	Red alert	Yellow alert
System‐related alert		
Pacing	Battery voltage decreased, reset, safety mode, battery EOL	Elective replacement indicator (ERI) and magnetic resonance imaging (MRI) mode
Shock/ATP treatment	Detection and treatment of ventricular fibrillation interrupted and charging time extended out of specification	
Data transmission		Deviation from specified transmission interval
Lead‐related alert		
Pacing	OOS threshold elevation of right ventricular lead, OOS resistance, lead polar switch in ventricular pacing‐dependent patients	OOS threshold elevation or OOS resistance of right ventricular lead or right atrial/left ventricular lead not mentioned on the left side
Shock/ATP treatment	ICD leads showing OOS shock resistance, pacing threshold, and resistance	
Syndrome‐related alert		
Atrial arrhythmia		OOS AHRE record or elevated heart rate episodes during arrhythmia
Ventricular arrhythmia		Increases in OOS non‐sustained ventricular tachycardia or ventricular extrasystoles in patients with impaired left ventricular function or heart failure
Heart failure‐related parameter		OOS heart failure‐related indices (including decreased activity or thoracic impedance, and device‐specific indices)
Ventricular pacing rate		OOS increases in the ventricular pacing rate in patients with sick sinus syndrome, impaired left ventricular function or heart failure
CRT		Sub‐standard biventricular pacing rate
Shock actuation	All shock actuations	
ATP actuation		All ATP actuations

*Note:* Red alerts indicate conditions requiring urgent or semi‐urgent response. Yellow alerts indicate events requiring early clinical review but not immediate intervention.

However, patients should be informed in advance that this is not an emergency response system, and patients should be contacted as necessary depending on the situation [53]. While each manufacturer offers nominal alert settings, the most fundamental function of RM is the continuous verification of the electrical parameters of CIEDs and leads—items that are routinely assessed during outpatient device follow‐ups as described above. Consistent with other guidelines [77], RM is particularly essential for patients with implanted devices or leads subject to recalls or safety advisories. Several studies have reported on RM's role in detecting issues such as premature battery depletion and lead failure [78, 79, 80, 81]. However, RM must also facilitate urgent or semi‐urgent responses to events such as device resets, transition to safety mode, and other critical alerts. Furthermore, with respect to ICDs, detection of ventricular fibrillation, treatment discontinuation, therapy delivery failures, and OOS prolongation of capacitor charging time may require an emergency or semi‐emergency response. Regarding leads, right ventricular lead threshold elevation/OOS resistance in pacing‐dependent patients, lead polarity switching, OOS shock impedance or right ventricular impedance in ICD leads, and noise sense episodes also require an emergency or semi‐emergency response. Accordingly, these items were listed as Class I recommendations.

Electrical storms leading to frequent ICD shocks are considered “medical emergencies” because of their association with significantly poor prognosis [91]. Furthermore, the results of the MADIT and MADIT‐RIT studies suggest that ICD shock deliveries, whether appropriate or inappropriate, are associated with adverse outcomes [92, 93]. In addition, inappropriate anti‐tachycardia pacing (ATP) therapy is also reported to result in poor prognosis, emphasizing the importance of appropriate alert settings in RM systems to minimize these events [70, 82, 83]. Such alert‐based monitoring enables timely emergency or semi‐emergency response, while also reducing unnecessary in‐person consultations without compromising follow‐up adherence [70]. Moreover, it has been reported to reduce the incidence of inappropriate therapies [15].

In recent years, heart failure monitoring has become possible using implanted devices such as pacemakers, ICDs, or CRT‐Ds. A pooled analysis of three studies using specific systems in ICD and CRT‐D patients showed improved clinical outcomes (all these three studies cited in this paper used Biotronik's Home Monitoring with a workflow that responds based on alerts being set in daily monitoring using multiple indicators, including activity trends) [86]. However, a meta‐analysis of data from multiple studies [84] has not yet confirmed a prognostic benefit compared with standard medical care. Furthermore, a study evaluating a single heart failure indicator failed to show a significant prognostic value [35]. Conversely, monitoring strategies that integrate multiple heart failure indicators [38, 83, 85] and ensure appropriate patient care after an alert have been shown to reduce heart failure–related hospitalizations and help identify patients with high‐risk prognoses [94]. As highlighted in the above‐mentioned pooled analysis [86], comprehensive monitoring that incorporates multiple parameters combined with timely, tailored patient management may effectively decrease heart failure hospitalizations and is particularly recommended for ICD and CRT‐D recipients at high risk of heart failure.

As part of the heart failure monitoring described above, many studies have shown that a higher biventricular pacing rate leads to improved outcomes in CRT‐implanted patients [95, 96, 97]. Although the optimal cutoff value remains a matter of debate, it is generally recommended to set an alert if the biventricular pacing rate falls below 90% [83, 86].

Since the publication of the ASSERT study [25], many studies have shown that atrial high‐rate episodes (AHRE) detected by CIEDs are associated with increased risk of embolic events [23, 87]. AHRE has also been reported to be linked to the development of heart failure through a reduction in CRT pacing rates [98] and monitoring of supraventricular arrhythmias has been shown to reduce inappropriate ICD therapies [15]. Several AHRE‐related parameters—such as episode heart rate, duration, and AF burden—have been investigated in relation to therapeutic decision‐making. Although the definitive cutoff value for intervention has not been determined, multiple guidelines consistently recommend routine monitoring of AHRE in patients with implanted devices [88, 89].

In patients with sick sinus syndrome who do not require ventricular pacing, a higher right ventricular pacing rate increases the risk of adverse events such as heart failure and AF [99]. Similarly, in ICD‐implanted patients with impaired left ventricular function, elevated right ventricular pacing rates have been linked to poorer clinical outcomes [100]. Although no studies have yet demonstrated that reducing right ventricular pacing rates through RM monitoring directly improves prognosis, several right ventricular pacing avoidance algorithms have been developed and shown to enhance outcomes. Accordingly, the 2021 European Society of Cardiology (ESC) guidelines recommend the use of such algorithms [77]. Implementing a right ventricular avoidance algorithm based on alert settings early after implantation may help prevent deterioration of cardiac function, and there have been reports of these strategies being successfully applied under RM [90].

### 8.2 Remote Monitoring Alert Setting Parameters Recommended for Patients With ILRs

ILRs are widely used in routine clinical practice for the detection of AF following cryptogenic stroke and for the evaluation of unexplained syncope, palpitations, and AF [101]. Their use is endorsed by many guidelines, particularly for these two clinical indications [42, 77, 102]. Table 20 summarizes the basic specifications of ILRs provided by each manufacturer. Although nominal alert settings are available for various parameters, false positive detections remain common [103]. For this reason, it is recommended to optimize alert settings using the RM programming function to reduce false positive events while avoiding the need for additional hospital visits or imposing unnecessary burdens on patients [56, 101].

**TABLE 20:** Basic performance of ILRs from various manufacturers.

Company	Abbott	Biotronik	Medtronic
Latest model	Assert‐IQ EL+	BIOMONITOR IIIm	LINQ II
Length	49.5 mm	77.5 mm	45.1 mm
Size	1.9 cc	1.9 cc	1.6 cc
Weight	3.7 g	4.0 g	4.0 g
Max lifespan	6.6 years	5.5 years	4.5 years
Max recording time	60 min	67 min	61 min (30 min in case of symptom)
Recorded episode type	5 (Symptom/AF/tachycardia/bradycardia/pause)	6 (Symptom/AF/HVR/SRD/bradycardia/asystole)	7 (Symptom/AF/AT/VT/FVT/SRD/bradycardia/pause)
Max number of episodes	Remote transmission of all episodes	60	100
Number of episodes per day	No Limitation	3 (4 only in case of symptom)	3
AF detection time	30 s–60 min*	30 s–30 min** (confirmation time)	2 min
AT differentiation	No	No	Yes
Other	Setting can be changed on the Internet (RM)	Setting can be changed for 10 parameters including AF sensitivity and detection window in each patient	Setting can be changed on the Internet (RM)

*Note:* Specifications are based on manufacturer information available as of 2024.

Abbreviations: AF, atrial fibrillation; AT, atrial tachycardia; FVT, fast ventricular tachycardia; VT, ventricular tachycardia.

Table 21 presents the recommended ILR alert settings, and Table 22 outlines the recommended ILR alert classifications, distinguishing between red alerts (requiring urgent attention) and yellow alerts (requiring non‐urgent review).

**TABLE 21:** Recommended ILR alert settings.

Recommendation classes	Evidence level	Recommendation
I	B‐NR	Adjust alert settings based on the underlying clinical indication for ILR implantation (e.g., cryptogenic stroke, unexplained syncope, or palpitations) [56, 101].
I	B‐NR	Adjust alert settings to ensure accurate diagnosis, as ILRs have a high incidence of false‐positive detections for arrhythmias [56, 101, 103].

*Note:* Alert settings should be adjusted according to the clinical indication for ILR implantation such as cryptogenic stroke or syncope evaluation. Colour‐coded in descending order of recommendation strength (green, yellow, pale yellow, orange) and evidence level (dark blue gradually lightening to pale blue).

**TABLE 22:** Recommended red alert and yellow alert settings for ILR.

	Red alert	Yellow alert
System‐related alert		
		Battery depletion, reset
Syndrome‐related alert		
Bradycardia	Atrioventricular block can be confirmed with bradycardia of 30/min or less, or asystole lasting 6 s or longer.	Atrioventricular block cannot be confirmed with bradycardia of 30/min or less, or asystole lasting 3 s or longer.
Tachycardia	Tachycardia of 230/min or more persists for 30 beats or longer.	Tachycardia with a rate of 180/min or higher lasting for 16 consecutive beats or more.
Atrial fibrillation	Atrial fibrillation lasting for 6 min or longer in patients with cryptogenic stroke	Atrial fibrillation lasting for 6 min or longer in patients other than those with cryptogenic stroke

*Note:* Alert classification reflects urgency of clinical response and may be modified according to institutional protocols and patient characteristics.

### 8.3 Synopsis

Alert‐based monitoring managed solely by automated RM offers the advantage of significantly reducing staff workload while still allowing for prompt response as necessary. Key alert items include battery life and lead failure settings, as well as tachycardia therapy settings for ICDs and changes in biventricular pacing rates for CRTs. For ILRs, optimizing alert settings is essential to improve diagnostic accuracy, as nominal settings often result in overdiagnosis of arrhythmias. Furthermore, heart failure monitoring that integrates multiple physiological markers is expected to enable early prediction of heart failure worsening and reduce the rate of rehospitalizations.

## 9 Pediatric Telemonitoring

As in adults, RM in pediatric patients (defined as patients under 21 years of age or managed by a pediatrician) offers the advantage of detecting and intervening on issues such as battery depletion and lead or device failure [104, 105]. A key characteristic of pediatric patients is that the younger they are, the more likely they are to experience lead and CIED malfunctions. Because RM facilitates the prompt detection of lead abnormalities shortly after implantation, early implementation is strongly recommended. It is also reported that tachyarrhythmia is the most frequently transmitted alert event [104]. Previous studies have found that the median RI interval was 91 days, and the median time from the last follow‐up to the occurrence of an event requiring intervention was 46 days [106]. Based on these findings, the appropriate RI interval is considered to be every 3–12 months for pacemakers and every 3–6 months for ICDs [104, 106]. As device replacement approaches, the frequency of RI should be increased, similar to adult practice [104]. While the recommendations described in this statement are also applicable to pediatric patients, the 2021 PACES statement provides specific guidance for RM in this population [105]. Pediatricians play a crucial role in fostering independence as patients transition from adolescence to adulthood, educating them about their medical condition, CIED function, and the importance of RM. While the transition process varies for each patient, open communication and shared medical information among pediatric patients, parents, and healthcare providers are essential for ensuring a smooth and effective transition to adult care [43, 105].

## 10 Conclusions

More than a decade has passed since RM became covered by health insurance. Based on the challenges that have arisen during this time, we have formulated a statement tailored to the current situation in Japan. The statement highlights four key points, summarized as follows:

When introducing RM, it is essential to register patients and confirm the connection at an early stage. It is also important to ensure that patients, families, and caregivers understand the benefits of RM and appropriate management methods through appropriate education to promote long‐term adherence. For ILR use in particular, RM should be initiated immediately after implantation, with programming and follow‐up protocols tailored to each patient's underlying disease.

To ensure the smooth implementation of RM, it is essential to allocate sufficient medical personnel in proportion to the number of patients and secure adequate time for the assigned workload. Medical staff should continually enhance their expertise and are encouraged to obtain CDR or JHRS Implantable Cardiac Arrhythmia Device Certification. It is necessary to form a multidisciplinary RM team to facilitate internal information sharing and to ensure that monitoring results are accurately documented in electronic medical records.

We recommend introducing alert‐based RM to address the increasing workload. Prompt responses to urgent alerts and device‐specific alert programming help reduce both the volume of transmitted data and the overall workload. In addition, leveraging third‐party support systems provides flexibility in managing workload fluctuations and helps prevent omissions in medical fee claims.

Finally, close cooperation between medical institutions and device manufacturers is essential, including the bidirectional exchange of ideas regarding training of medical professionals, patient education, and the enhancement of patient care services. Device manufacturers are also expected to respond promptly and accurately to recalls and safety advisories and to continuously improve the reliability and safety of their products.

## 11 Future Prospects

Looking ahead, the following three challenges can be identified:

Adapting to evolving technology: With the ongoing advancement of digital and device technologies, new forms of RM technologies are expected to emerge. Accordingly, this statement will need to be periodically updated and expanded to reflect these technological developments and ensure continued relevance.

Enhancing international collaboration: Strengthening global collaboration and information exchange will help deepen understanding of RM practices in Japan and contribute to the development of more effective and internationally aligned guidance.

Promoting patient and caregiver engagement: Active involvement of patients and caregivers is crucial for achieving optimal treatment outcomes. It is therefore important to establish systems and educational resources that encourage patients to take an active role in RM and to support their long‐term engagement.

## Conflicts of Interest

The COI disclosures in preparing this statement are as follows: E.W. is an Advisor of Fukuda Denshi. N.N. is affiliated with the endowed department by Japan Medtronic Inc. N.N. has received honoraria for lectures from Medtronic, Cook, Philips, and Boston Scientific. K.F. is affiliated with an endowed department supported by Biotronik, Boston Scientific, Medtronic, and Simplex Quantum. R.K. receives lecture fees from Boston Scientific and scholarship donations from Japan Lifeline for his clinical department. T.N. received honoraria/speakers' bureaus from Medtronic, Abbott, and Biotronik outside of the submitted work. H.T. received honoraria for lectures or speakers bureaus from DAIICHI SANKYO COMPANY Ltd., Medtronic Japan Co. Ltd., BIOTRONIK Japan Inc., and Boston Scientific Japan K.K. H.T. received grants (Investigator initiated study unrelated to manuscript topic) from Abbott Medical Japan LLC.

## Data Availability

Data sharing not applicable to this article as no datasets were generated or analysed during the current study.
